# Association between the triglyceride–glucose index and acute kidney injury in patients undergoing percutaneous coronary: a retrospective analysis of the MIMIC-IV database

**DOI:** 10.1186/s13098-025-01647-2

**Published:** 2025-03-03

**Authors:** Fan Zhang, Shen Zhan, Lihong Zhang, Xin Zheng, Xiangru Li, Yuzhu Wang

**Affiliations:** Department of Nephrology, Haidian Hospital (Haidian Section of Peking University Third Hospital), Beijing, 100191 China

**Keywords:** Triglyceride–glucose index, Acute kidney injury, Percutaneous coronary intervention, Diabetes mellitus

## Abstract

**Background:**

Acute kidney injury (AKI) is a common complication that affects the outcomes of patients undergoing percutaneous coronary intervention (PCI). The triglyceride–glucose (TyG) index, a metric computed from fasting blood triglyceride and glucose levels, is closely associated with poor PCI outcomes. This study examined the association between the TyG index and incidence of AKI in patients undergoing PCI.

**Methods:**

Clinical information was obtained from the Medical Information Mart for Intensive Care IV database, which contains clinical data on 70,000 patients admitted to the intensive care unit at Beth Israel Deaconess Medical Center from 2008 to 2019. In total, 435 patients who underwent PCI were enrolled in this retrospective study, and they were categorized according to their AKI status, TyG quartiles, and diabetes mellitus (DM) history to analyze their baseline characteristics. The association of the TyG index with the risk of AKI was assessed using restricted cubic spline regression and logistic regression models. Subgroup analyses were also performed in patients with and without DM.

**Results:**

Compared with the non-AKI population, patients with AKI who underwent PCI had a higher mean TyG index (p = 0.004). The restricted cubic spline regression model revealed a linear correlation between the TyG index and AKI risk (p for nonlinear = 0.123) in patients undergoing PCI. A high TyG index was a risk factor for AKI in non-DM subgroup, as well as in patients with BMI < 28 (odds ratio [OR] = 1.77; p = 0.050) and those with no history of diabetes (OR = 1.83; p = 0.047) or COPD (OR = 1.56; p = 0.030).

**Conclusions:**

This study highlighted the role of the TyG index as a predictive biomarker for AKI in patients without DM undergoing PCI, providing clinicians with a tool for identifying high-risk individuals for early intervention.

**Supplementary Information:**

The online version contains supplementary material available at 10.1186/s13098-025-01647-2.

## Introduction

Percutaneous coronary intervention (PCI) is the primary treatment for acute coronary syndrome in patients with coronary artery disease (CAD), and it is linked to high efficacy and low surgical risk [1]. Despite improvements in devices and increased procedural safety, PCI-related complications, including catheter-related complications and procedural complications, remain unavoidable [2]. Acute kidney injury (AKI) is the most common complication after PCI, occurring at an incidence of approximately 3–20%, and it severely affects the quality of PCI treatment [3]. In addition to leading to longer hospital stay and bleeding, PCI-related AKI is involved in the occurrence of myocardial infarction, persistent renal impairment, and even periprocedural mortality [4]. Fortunately, PCI-related AKI can be avoided with effective clinical interventions, such as adequate hydration. Nevertheless, the risk factors for AKI in patients undergoing PCI remain unclear, hampering early prediction and intervention to improve prognosis.

The triglyceride–glucose (TyG) index, derived from the fasting blood glucose (FBG) and triglyceride (TG) concentrations, has been linked to insulin resistance and the occurrence of CAD [5]. Insulin resistance is a pivotal mechanism contributing to the development of type 2 diabetes mellitus (T2DM), an acknowledged risk factor for CAD progression [6]. As recently reported, the TyG index is associated with poor prognosis in patients with CAD or ischemic heart failure after PCI [7, 8]. In addition, the TyG index has been identified as an independent and causal risk factor for heart failure [9]. In addition, it is positively correlated with the risk of coronary heart disease, and it reflects the severity of coronary atherosclerosis [10]. In terms of kidney diseases, the TyG index increases the risk of chronic kidney disease independently of established risk factors [11]. Renal function plays a key role in mediating the relationship between the TyG index and cardiovascular risk [12]. However, the effect of the TyG index on the risk of AKI in patients undergoing PCI has not been analyzed.

Therefore, this study evaluated whether a high TyG index increased the risk of AKI in patients after PCI and in other subgroups such as patients with or without DM. We aimed to identify novel factors predictive of the risk of AKI after PCI to improve the outcomes and quality of life of patients.

## Materials and methods

### Study design

In this retrospective observational study, data were obtained from the Medical Information Mart for Intensive Care IV (MIMIC-IV) database (version 2.2, https://physionet.org/content/mimiciv/2.2/). The dataset, comprising more than 70,000 intensive care unit (ICU) admissions at Beth Israel Deaconess Medical Center (BIDMC) from 2008 to 2019, was ethically approved by the Institutional Review Boards of both the Massachusetts Institute of Technology and BIDMC. Database access was granted to the author Fan Zhang under the approval number 66880658. The design of MIMIC-IV has been published previously [13].

Data extraction was performed using Navicat Premium 16. The study included ICU-admitted adults diagnosed with atherosclerotic heart disease of the native coronary artery based on the Ninth and Tenth Revisions of the International Classification of Diseases. Patients who underwent PCI were identified using procedure long titles such as “coronary artery stent” and “dilation of coronary artery.” Further filtering was applied to isolate the records of patients undergoing PCI for the first time based on admission timelines.

**Fig. 1:**
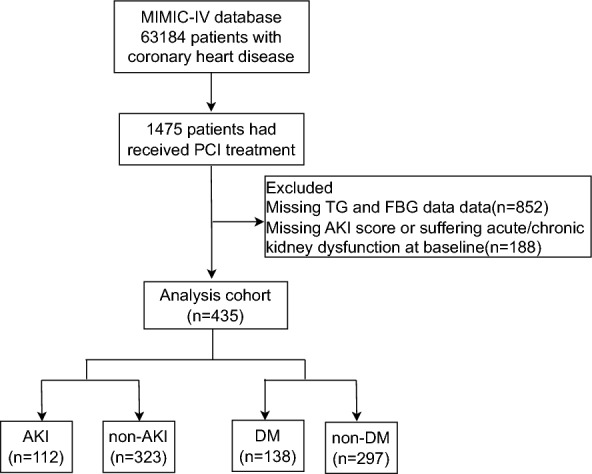
Flowchart of patient selection

The MIMIC-IV database includes 63,184 patients with coronary heart disease, 1475 of whom underwent PCI. The inclusion criteria were as follows: age ≥ 18 years; diagnosis of atherosclerotic heart disease of the native coronary artery based on the Ninth and Tenth Revisions of the International Classification of Diseases, including ST-segment elevation myocardial infarction (STEMI), non-STEMI, and unstable angina; and receipt of PCI during hospitalization. The exclusion criteria were as follows: acute/chronic kidney dysfunction on ICU admission and missing serum creatinine, TG, and/or FBG data on ICU admission. AKI was defined according to the Kidney Disease: Improving Global Outcomes (KDIGO) guidelines as an increase in the serum creatinine level of at least 0.3 mg/dL within 48 h or an increase in the serum creatinine level of 1.5-fold versus baseline within 7 days. The patient screening flow chart is displayed in Fig. 1.

### Data extraction

Data on baseline characteristics within first the 24 h of ICU admission before PCI were extracted from the MIMIC-IV database. The extracted data included the following categories: demographic information including age, sex, height, and weight; laboratory parameters including white blood cell (WBC) and platelet counts, hemoglobin, platelet, FBG, serum sodium, serum potassium, serum calcium, serum phosphate, TG, high-density lipoprotein (HDL), low-density lipoprotein (LDL), and cholesterol levels, renal function, and the estimated glomerular filtration rate; comorbidities including hypertension, diabetes, chronic obstructive pulmonary disease (COPD), peripheral vascular disease, stroke, cerebral hemorrhage, and AKI; and medication consisting of statins and antiplatelet agents. The TyG index was calculated as Ln (fasting TGs [mg/dL] × FBG [mg/dL]/2). AKI was diagnosed using the KDIGO criteria, whereas other comorbidities were identified using International Classification of Diseases codes.

### Outcome

The primary outcome in the present study was the incidence of AKI after PCI.

### Statistical analysis

Categorical variables were analyzed using the chi-squared test. Non-normally distributed continuous variables were presented as the median (lower quartile, upper quartile). Normally distributed continuous variables were presented as the mean ± standard deviation. The Kruskal–Wallis nonparametric test or one-way ANOVA was used to analyze the differences in numerical variables between the groups.

To examine the association between the TyG index and risk of AKI, multivariable logistic regression was used. The TyG index was analyzed as a continuous variable, as quartiles (using Q1 as the reference group), and as a non-linear function using a restricted cubic spline (RCS) function with five internal knots (5th, 27.5th, 50th, 72.5th, and 95th percentiles of TyG in the study population) with the median (TyG index of 9) as a reference. Model 1 was unadjusted, whereas model 2 was adjusted for age and sex. Model 3 was adjusted for model 2 variables plus BMI, SBP, DBP, hemoglobin, platelet, WBC counts, creatinine, and BUN. Model 4 was adjusted for model 2 variables plus a history of hypertension, DM, COPD, peripheral vascular disease, and stroke. The selection of covariates was primarily based on previous studies [14, 15] and clinical expertise. The odds ratio (OR) and 95% confidence interval (CI) were reported for each model, along with the corresponding p-value.

Then, we conducted subgroup analyses, including intergroup comparisons, RCS, and logistic regression modeling. Additional subgroup analyses were performed according to the following risk factors: sex, age (< 65 years or ≥ 65 years), BMI (< 28 or ≥ 28), DM, hypertension, and COPD. The OR and 95% CI for the continuous variable of TyG and the four quantiles were reported for each risk factor subgroup.

All data were analyzed using SAS 9.4 and R 4.4.2 software. Two-sided p < 0.05 indicated significance.

## Results

### Baseline characteristics of patients grouped by AKI

In this study, 852 patients were excluded because of missing TG and FBG data on ICU admission, and 188 were eliminated because of missing serum creatinine data or the presence of acute/chronic kidney dysfunction on ICU admission, leaving 435 patients in the analysis. This group included 138 and 297 patients in the DM and non-DM groups, respectively. In addition, 112 patients were diagnosed with AKI, and the remaining 323 patients comprised the non-AKI group. Comparisons of the groups are presented in Table 1. Compared with the non-AKI group, the AKI had a higher mean TyG index (p = 0.004); older age (p = 0.026); lower DBP (p = 0.003), higher rates of diabetes (p < 0.001) and stroke (p = 0.016); higher WBC counts (p = 0.004); higher potassium (p = 0.011), glucose (p < 0.001), and phosphate levels (p < 0.001); lower HDL (p < 0.001), LDL (p < 0.001), cholesterol (p < 0.001), calcium (p < 0.001), hemoglobin (p < 0.001), and statin levels (p = 0.027); and a lower AKI score (p < 0.001). No significant differences were observed in parameters such as sex, BMI, SBP, and rates of hypertension between the two groups (Table 1).

**Table 1:** Baseline characteristics according to AKI occurrence

Characteristics	Overall n = 435	AKI n = 112	Non-AKI n = 323	P value
TyG, mean ± sd	9.11 ± 0.71	9.3 ± 0.84	9.04 ± 0.65	0.004
Q1	108 (24.83)	20 (17.86)	88 (27.24)	0.001
Q2	108 (24.83)	20 (17.86)	88 (27.24)	
Q3	109 (25.06)	29 (25.89)	80 (24.77)	
Q4	109 (25.06)	20 (17.86)	89 (27.55)	
Age, mean ± sd	67.14 ± 13.72	69.63 ± 13.49	66.28 ± 13.71	0.026
Sex				0.802
Male	291 (66.9)	76 (67.86)	215 (66.56)	
Female	144 (33.1)	36 (32.14)	108 (33.44)	
BMI, kg/m^2^, mean ± sd	28.76 ± 5.76	28.93 ± 5.54	28.7 ± 5.85	0.710
Sbp, mmHg, mean ± sd	125.73 ± 21.69	122.15 ± 25.3	126.97 ± 20.18	0.069
Dbp, mmHg, mean ± sd	73.42 ± 16.15	69.53 ± 16.72	74.77 ± 15.75	0.003
Hypertension	296 (68.05)	82 (73.21)	214 (66.25)	0.173
Diabetes	138 (31.72)	55 (49.11)	83 (25.7)	< 0.001
COPD	34 (7.82)	13 (11.61)	21 (6.5)	0.083
Peripheral vascular disease	9 (2.07)	3 (2.68)	6 (1.86)	0.599
Stroke	18 (4.14)	9 (8.04)	9 (2.79)	0.016
Cerebral hemorrhage	3 (0.69)	2 (1.79)	1 (0.31)	0.104
Sodium, mEq/L, mean ± sd	137.93 ± 3.47	137.54 ± 4.35	138.06 ± 3.11	0.168
Potassium, mEq/L, median (Q1, Q3)	4.1 (3.8, 4.4)	4.2 (3.9, 4.6)	4.1 (3.8, 4.4)	0.011
Calcium, mg/dL, mean ± sd	8.65 ± 0.72	8.38 ± 0.96	8.74 ± 0.6	< 0.001
Phosphate, mg/dL, mean ± sd	3.54 ± 1.03	3.86 ± 1.37	3.42 ± 0.86	< 0.001
Ast, IU/L, median (Q1, Q3)	92.33 (52, 160)	114.5 (44, 280.5)	90.84 (54.6, 145)	0.075
Alt, IU/L, median (Q1, Q3)	42.86 (28.46, 59)	44.76 (26, 102.5)	42.8 (29.93, 56.71)	0.183
Hemoglobin, g/dL, mean ± sd	12.64 ± 2.07	11.92 ± 2.23	12.89 ± 1.96	< 0.001
Platelet, K/uL, mean ± sd	233.01 ± 81.66	228.42 ± 97.65	234.6 ± 75.44	0.543
WBC, K/uL, median (Q1, Q3)	10.8 (8.4, 13.9)	12.05 (8.55, 16.95)	10.6 (8.3, 13.2)	0.004
HDL, mg/dL, median (Q1, Q3)	41.72 (34, 50)	36.5 (26, 48)	42 (36, 51)	< 0.001
LDL, mg/dL, median (Q1, Q3)	92 (66, 118)	74 (51, 98.93)	98 (74, 124)	< 0.001
Triglycerides, mg/dL, median (Q1, Q3)	119 (87, 176)	120.5 (85, 178)	119 (88, 176)	0.856
Cholesterol, mg/dL, median (Q1, Q3)	164 (132, 194)	136 (111.57, 169.68)	171 (139.03, 198)	< 0.001
Glucose, mg/dL, mean ± sd	155.81 ± 75.03	193.75 ± 105.31	142.66 ± 55.6	< 0.001
Statin	424 (97.47)	106 (94.64)	318 (98.45)	0.027
Antiplatelet	434 (99.77)	112 (100)	322 (99.69)	0.556
AKI score after PCI				
0	323 (74.25)	0 (0)	323 (100)	< 0.001
1	67 (15.40)	67 (59.82)	0 (0)	
2	19 (4.37)	19 (16.96)	0 (0)	
3	26 (5.98)	26 (23.21)	0 (0)	

According to the TyG index quartiles, we classified the patients into four groups. As displayed in Supplementary Table 1, a higher TyG index was generally associated with younger age (p = 0.017); higher BMI (p = 0.036); a higher prevalence of diabetes (p < 0.001); lower sodium levels (p = 0.010); higher phosphate (p = 0.001), hemoglobin (p = 0.023), TG (p < 0.001), cholesterol (p = 0.011), and glucose levels (p < 0.001); lower HDL levels (p < 0.001); higher WBC counts (p < 0.001); and a higher AKI score (p = 0.005).

When grouped by DM history, the DM group had a higher TyG index (p < 0.001); older age (p < 0.001); a higher proportion of women (p = 0.041); lower DBP (p < 0.001); a higher prevalence of hypertension (p < 0.001); and a higher incidence of peripheral vascular disease (p = 0.003), stroke (p = 0.001), and cerebral hemorrhage (p = 0.011, Supplementary Table 2). Laboratory parameters such as sodium (p = 0.004), calcium (p = 0.044), phosphate (p < 0.001), AST (p < 0.001), ALT (p = 0.002), hemoglobin (p < 0.001), HDL (p < 0.001), LDL (p < 0.001), TG (p = 0.0026), cholesterol (p < 0.001), and glucose levels (p < 0.001) significantly differed between the DM and non-DM groups.

### Correlation between the TyG index and the risk of AKI

**Fig. 2:**
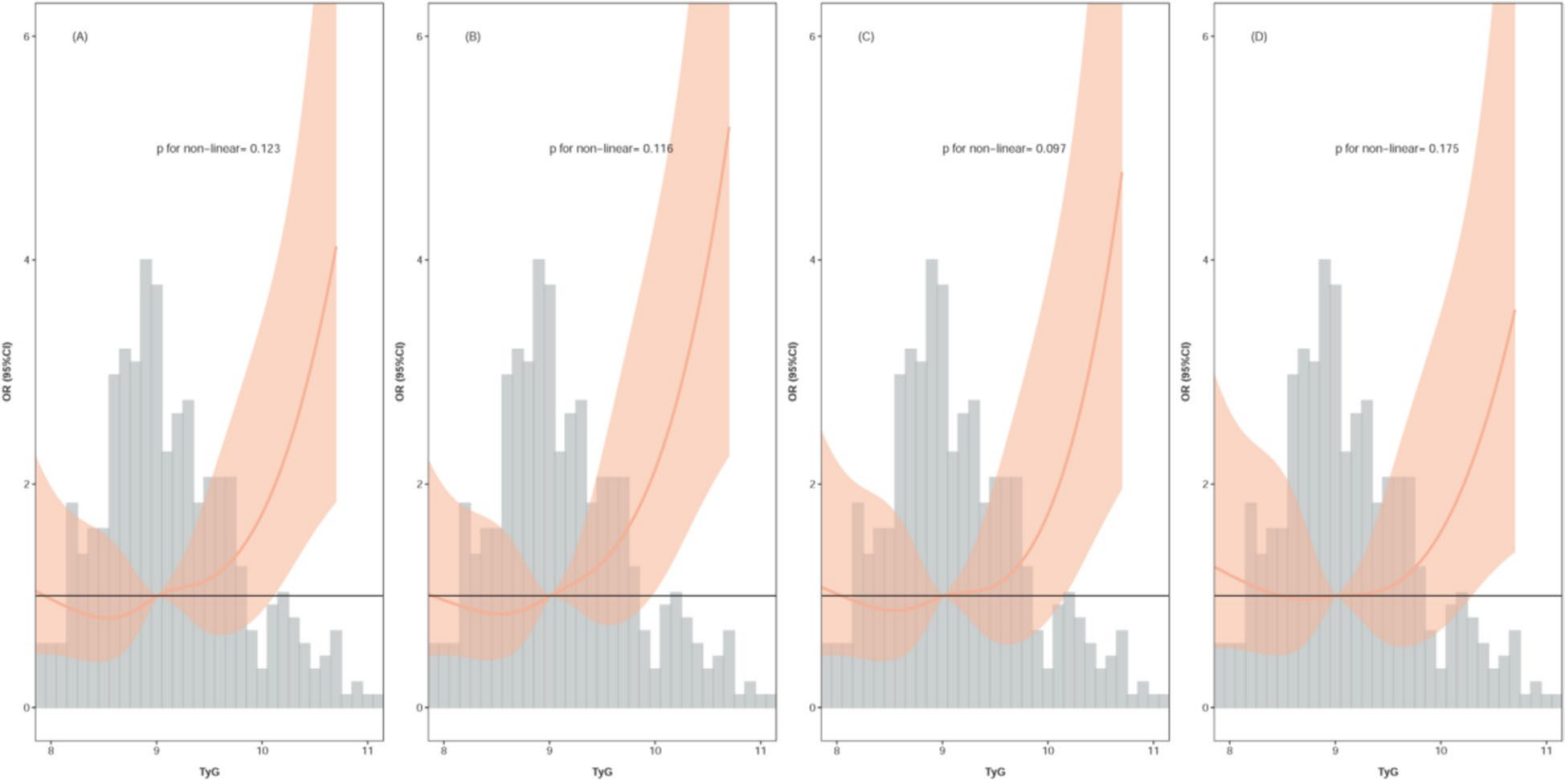
The correlation between the TyG index and occurrence of AKI in the RCS population. **A** Model 1; **B** model 2; **C** model 3; **D** model 4

The RCS curve illustrated in Fig. 2 indicates a linear association between the TyG index and the risk of AKI in models 1 (p for nonlinear = 0.123) and 2 (p for nonlinear = 0.116). A linear association was also observed in models 3 (p for nonlinear = 0.097) and 4 (p for nonlinear = 0.175).

### Association between the TyG index and the risk of AKI in all participants and in the non-DM subgroup

As presented in Table 2, the CPH model confirmed that the TyG index was a notable risk factor for AKI in patients undergoing PCI in models 1 (OR = 1.66; 95% CI = 1.22–2.26; p = 0.001), 2 (OR = 1.83; 95% CI = 1.33–2.53; p < 0.001), and 3 (OR = 1.47; 95% CI = 1.33–2.53; p = 0.041). Moreover, the risk of AKI tended to increase as the TyG index increased. Patients in Q4 of the TyG index had a particularly high AKI risk in models 1 (OR = 2.87; 95% CI = 1.54–5.32; p = 0.001), 2 (OR = 3.46; 95% CI = 1.82–6.58; p < 0.001), and 3 (OR = 2.37; 95% CI = 1.12–5.03; p = 0.025).

**Table 2:** Association of TyG indicators and AKI, among overall participants and DM subgroups

	Model1		Model2		Model3		Model4	
	OR (95% CI)	P value	OR (95% CI)	P value	OR (95% CI)	P value	OR (95% CI)	P value
*Overall*								
TyG, per unit	1.66 (1.22,2.26)	0.001	1.83 (1.33,2.53)	< 0.001	1.47 (1.02,2.12)	0.041	1.16 (0.78,1.71)	0.469
*Tyg*								
Q1	Ref.		Ref.		Ref.		Ref.	
Q2	1.59 (0.84,3.04)	0.156	1.65 (0.86,3.17)	0.135	1.58 (0.76,3.29)	0.216	1.34 (0.63,2.84)	0.444
Q3	0.99 (0.5,1.96)	0.974	1.02 (0.51,2.05)	0.946	0.69 (0.31,1.53)	0.362	0.49 (0.21,1.16)	0.103
Q4	2.87 (1.54,5.32)	0.001	3.46 (1.82,6.58)	< 0.001	2.37 (1.12,5.03)	0.025	1.5 (0.66,3.37)	0.332
*Non DM subgroup*								
TyG, per unit	1.79 (1.13,2.84)	0.013	2.03 (1.25,3.29)	0.004	1.87 (1.05,3.34)	0.034	1.83 (1.01,3.32)	0.047
*Tyg*								
Q1	Ref.		Ref.		Ref.		Ref.	
Q2	1.42 (0.64,3.15)	0.393	1.49 (0.66,3.34)	0.333	1.8 (0.73,4.47)	0.202	1.87 (0.72,4.82)	0.197
Q3	0.98 (0.41,2.35)	0.957	1.09 (0.45,2.64)	0.857	1.04 (0.38,2.87)	0.942	1.03 (0.36,2.91)	0.963
Q4	2.93 (1.31,6.52)	0.009	3.68 (1.58,8.57)	0.003	3.53 (1.24,10.04)	0.018	3.56 (1.21,10.52)	0.021
*DM subgroup*								
TyG, per unit	1.07 (0.68,1.68)	0.770	1.13 (0.7,1.82)	0.611	1.14 (0.68,1.92)	0.619	1.09 (0.64,1.85)	0.747
*Tyg*								
Q1	Ref.		Ref.		Ref.		Ref.	
Q2	0.78 (0.21,2.94)	0.711	0.82 (0.21,3.16)	0.769	0.61 (0.14,2.73)	0.522	0.7 (0.15,3.2)	0.649
Q3	0.34 (0.09,1.32)	0.120	0.32 (0.08,1.24)	0.099	0.21 (0.05,0.93)	0.040	0.21 (0.04,0.98)	0.047
Q4	0.83 (0.24,2.91)	0.775	0.95 (0.27,3.42)	0.943	0.74 (0.18,3.03)	0.673	0.72 (0.17,3.01)	0.653

Model 1: unadjusted model; Model 2: adjusting age and sex; Model 3: additionally adjusting BMI, SBP, DBP, hemoglobin, platelet, WBC, creatinine and BUN; Model 4: additionally adjusting history of hypertension, diabetes, COPD, peripheral vascular disease and stroke

Among patients without DM, the TyG index was a more pronounced risk factor for AKI in models 1 (OR = 1.79; 95% CI = 1.13–2.84; p = 0.013), 2 (OR = 2.03; 95% CI = 1.25–3.29; p = 0.004), 3 (OR = 1.87; 95% CI = 1.05–3.34; p = 0.034), and 4 (OR = 1.83; 95% CI = 1.01–3.32; p = 0.047). The patients in Q4 had a notably higher risk of AKI in models 1 (OR = 2.93; 95% CI = 1.31–6.52; p = 0.009), 2 (OR = 3.68; 95% CI = 1.58–8.57; p = 0.003), 3 (OR = 3.53; 95% CI = 1.24–10.04; p = 0.018), and 4 (OR = 3.56; 95% CI = 1.21–10.52; p = 0.021). By contrast, no correlation between the TyG index and the risk of AKI was found in the DM subgroup in any of the four models (p > 0.05).

### Impact of a high TyG index on the risk of AKI in different subgroups

Next, we analyzed the correlation between the TyG index and the occurrence of AKI in diverse populations grouped by sex, age, BMI, hypertension, diabetes, and COPD. As illustrated in Table 3, a high TyG index was a risk factor for AKI in patients with BMI < 28 (OR = 1.77; 95% CI = 1–3.15; p = 0.050) and those with no history of DM (OR = 1.83; 95% CI = 1.01–3.32; p = 0.047) or COPD (OR = 1.56; 95% CI = 1.04–2.34; p = 0.030). Nevertheless, no statistical difference was indicated by p for interaction.

**Table 3:** Subgroup analysis of association between TyG and AKI

Subgroups	Num. of AKI/Num. of participants (Proportion)	OR (95% CI)	p value	p for interaction
Sex				0.868
Male	76/291 (0.26)	1.5 (0.94,2.41)	0.092	
Female	36/144 (0.25)	1.48 (0.77,2.84)	0.241	
Age				0.480
> = 65	73/248 (0.29)	1.3 (0.78,2.17)	0.308	
< 65	39/187 (0.21)	1.74 (0.9,3.39)	0.100	
BMI				0.768
> = 28	64/232 (0.28)	1.2 (0.71,2.03)	0.490	
< 28	48/203 (0.24)	1.77 (1,3.15)	0.050	
Hypertension				0.899
Yes	82/296 (0.28)	1.39 (0.86,2.24)	0.180	
No	30/139 (0.22)	1.29 (0.66,2.54)	0.4547	
Diabetes				0.117
Yes	55/138 (0.40)	1.09 (0.64,1.85)	0.747	
No	57/297 (0.19)	1.83 (1.01,3.32)	0.047	
COPD				0.260
Yes	13/34 (0.38)	0 (0,4.41)	0.057	
No	99/401 (0.25)	1.56 (1.04,2.34)	0.030	

## Discussion

CAD is a serious public health hazard globally, and PCI is the acknowledged evidence-based therapy for CAD. Nevertheless, AKI is a common complication affecting the efficacy of PCI. Therefore, early prediction of AKI risk is indispensable for prevention by permitting timely and effective intervention. In this study, the clinical data of 435 patients who had undergone PCI were obtained from the public database MIMIC-IV and used to comprehensively analyze the association between the TyG index and the risk of AKI. Our results illustrated that a high TyG index was a risk factor for AKI in patients without DM undergoing PCI. These findings suggest that the TyG index represents an efficient biomarker in this population, offering clinicians a valuable reference for implementing appropriate and timely interventions.

The TyG index is a biomarker for identifying insulin resistance, a leading cause of elevated TG and glucose levels [16]. Our data demonstrated that a higher TyG index was more common in patients with DM. These results reflect the close relationships among the TyG index, insulin resistance, and DM, confirming the role of TyG in predicting insulin resistance and DM. In addition, a positive relationship between the TyG index and a higher prevalence of symptomatic CAD has been reported [17]. Furthermore, the TyG index is an independent predictor of major adverse cardiovascular events in patients with chronic and acute coronary syndrome after PCI [7, 18]. Research concerning the correlations of the TyG index with kidney diseases has primarily focused on chronic kidney diseases [19], but the influence of the TyG index on the incidence of AKI should be urgently elucidated.

The etiology of PCI-related AKI is complex, and surgical factors, contrast, and cardiac insufficiency can cause renal dysfunction [20]. Although a higher TyG index is associated with a higher risk of mortality in critically ill patients with AKI [21], the influence of the TyG index on AKI development has not been sufficiently explored. The present study indicated that among patients with CAD undergoing PCI, those with AKI had a higher mean TyG index than those without AKI. We also noted that the TyG index had a linear correlation with the risk of AKI, and a high TyG index was a risk factor for AKI development. This is similar to the viewpoint that the TyG index is an independent risk factor for contrast-induced AKI in patients with T2DM after coronary angiography [22]. Previous research found that a high TyG index is correlated with AKI risk in patients in the ICU [23]. However, this study demonstrated the relationship between the TyG index and incidence of AKI in patients without DM undergoing PCI for the first time. Meanwhile, evidence [24] suggests that emergency PCI is associated with a higher risk of AKI than elective PCI. Further prospective studies on the role of TyG in the occurrence of AKI in patients undergoing emergency and elective PCI are needed. Our study also analyzed the association between the TyG index and likelihood of AKI in different subpopulations grouped by BMI and histories of DM and COPD, providing a reference for personalized prevention in distinct populations.

The mechanism by which the TyG index increases the risk of AKI can be explained by the fact that a high TyG index is closely correlated with insulin resistance and DM, which are independent risk factors for AKI [25, 26]. Insulin resistance and related hyperinsulinemia are correlated with oxidative stress, which results in glomerular endothelial cell injury, mesangial cell proliferation, and basement membrane thickening [14]. This is supported by research reporting that severe β-cell dysfunction in young T2DM is associated with intraglomerular hypertension and kidney hyperoxia [27]. Moreover, individuals with a high TyG index are prone to unhealthy lifestyles and accompanying diseases such as hypertension and stroke, which are potential risk factors for AKI.

However, our data revealed that a high TyG index was a risk factor for AKI in patients without DM and those with BMI < 28. These findings could be attributable to the phenomenon of more serious metabolic disorders in patients with a higher TyG index despite no history of DM, which leads to more severe AKI in these patients. It was recently reported that an increased TyG index is associated with poor prognosis in patients with acute exacerbation of COPD [28]. However, our results demonstrated that a high TyG index was a risk factor for AKI in patients with no history of COPD; therefore, further research is needed regarding the associations among the TyG index, AKI, and COPD.

### Study limitations

To optimize future research, the limitations of this study should be addressed. First, a causal relationship could not be elucidated because of the retrospective and observational study design. Second, the data were derived from the MIMIC-IV database, which carries certain limitations, such as the lack of precise information about the type of PCI (emergency vs. elective). Therefore, data bias resulting from the unaccounted confounding factors was unavoidable. Further prospective studies are needed to confirm our conclusions.

## Conclusions

This study underscored the utility of the TyG index as a predictive biomarker for AKI in patients without DM undergoing PCI, providing clinicians with a tool to identify high-risk individuals for early intervention. Further prospective studies are required to confirm our findings.

## Supplementary Information

Supplementary Material 1.

Supplementary Material 2.

## Data Availability

No datasets were generated or analysed during the current study.
