# Supplementary material for: Association between the triglyceride–glucose index and acute kidney injury in patients undergoing percutaneous coronary: a retrospective analysis of the MIMIC-IV database
Source: Diabetol Metab Syndr. 2025 Mar 3;17:74. doi: 10.1186/s13098-025-01647-2 (PMC11874802; doi:10.1186/s13098-025-01647-2)
Supplement: Supplementary file 1 — Supplementary Material 1. [file 13098_2025_1647_MOESM1_ESM.docx]

# STable 1. Baseline characteristics by TyG quartile values

| **Characteristics** | **Overall**  **N=435** | **Q1**  **N=108** | **Q2**  **N=109** | **Q3**  **N=109** | **Q4**  **N=109** | **P value** |
| --- | --- | --- | --- | --- | --- | --- |
| TyG, mean±sd | 9.11±0.71 | 8.28±0.39 | 8.85±0.11 | 9.25±0.14 | 10.04±0.43 | <0.001 |
| Age, mean±sd | 67.14±13.72 | 68.97±15.37 | 68.74±13.65 | 67.16±13.42 | 63.72±11.76 | 0.017 |
| Sex |  |  |  |  |  |  |
| Male | 291(66.9) | 71(65.74) | 68(62.39) | 80(73.39) | 72(66.06) | 0.365 |
| Female | 144(33.1) | 37(34.26) | 41(37.61) | 29(26.61) | 37(33.94) | . |
| BMI,kg/m^2^, mean±sd | 28.76±5.76 | 27.87±5.7 | 28.36±6.39 | 28.75±5.24 | 30.05±5.52 | 0.036 |
| Sbp,mmHg, mean±sd | 125.73±21.69 | 123.16±19.01 | 126.11±21.82 | 125.74±21.79 | 127.89±23.88 | 0.452 |
| Dbp,mmHg, mean±sd | 73.42±16.15 | 73.44±14.41 | 73.45±16.96 | 73.69±17.27 | 73.11±16 | 0.995 |
| Hypertension | 296(68.05) | 63(58.33) | 78(71.56) | 81(74.31) | 74(67.89) | 0.064 |
| Diabetes | 138(31.72) | 12(11.11) | 32(29.36) | 39(35.78) | 55(50.46) | <0.001 |
| COPD | 34(7.82) | 10(9.26) | 8(7.34) | 7(6.42) | 9(8.26) | 0.880 |
| Peripheral vascular disease | 9(2.07) | 1(0.93) | 4(3.67) | 3(2.75) | 1(0.92) | 0.386 |
| Stroke | 18(4.14) | 1(0.93) | 6(5.5) | 3(2.75) | 8(7.34) | 0.083 |
| Cerebral hemorrhage | 3(0.69) | 0(0) | 0(0) | 2(1.83) | 1(0.92) | 0.299 |
| Sodium,mEq/L, mean±sd | 137.93±3.47 | 138.4±3.15 | 138.54±3.25 | 137.22±3.79 | 137.55±3.54 | 0.010 |
| Potassium,mEq/L,median(Q1,Q3) | 4.1(3.8, 4.4) | 4.1(3.7, 4.4) | 4.1(3.9, 4.4) | 4.1(3.8, 4.4) | 4.2(3.9, 4.5) | 0.100 |
| Calcium,mg/dL, mean±sd | 8.65±0.72 | 8.7±0.57 | 8.75±0.63 | 8.56±0.77 | 8.6±0.88 | 0.167 |
| Phosphate,mg/dL, mean±sd | 3.54±1.03 | 3.25±0.77 | 3.67±1.04 | 3.47±1.05 | 3.76±1.16 | 0.001 |
| Ast,IU/L,median(Q1,Q3) | 92.33(52, 160) | 88.19(53.48, 153.4) | 95.1(50, 155) | 84.04(50, 142) | 101.28(59.87, 213) | 0.356 |
| Alt,IU/L,median(Q1,Q3) | 42.86(28.46, 59) | 41.88(28.23, 56.85) | 40.68(28, 54) | 44.32(28.23, 59.64) | 47(32.36, 85.19) | 0.056 |
| Hemoglobin,g/dL, mean±sd | 12.64±2.07 | 12.41±1.89 | 12.27±2.12 | 12.94±1.95 | 12.94±2.25 | 0.023 |
| Platelet,K/uL, mean±sd | 233.01±81.66 | 229.48±72.85 | 232.47±77.29 | 231.75±78.08 | 238.31±97.09 | 0.876 |
| Wbc,K/uL,median(Q1,Q3) | 10.8(8.4, 13.9) | 10.1(7.9, 11.8) | 10(7.8, 13) | 11.9(8.7, 13.9) | 11.8(8.9, 15.5) | <0.001 |
| Creatinine,mg/dL,median(Q1,Q3) | 1(0.8, 1.2) | 0.9(0.8, 1.2) | 0.9(0.8, 1.2) | 1(0.8, 1.3) | 1.1(0.8, 1.4) | 0.081 |
| HDL,mg/dL,median(Q1,Q3) | 41.72(34, 50) | 47(40, 58) | 42(34, 51) | 40(34, 48) | 38.89(31, 43) | <0.001 |
| LDL,mg/dL,median(Q1,Q3) | 92(66, 118) | 88(63.5, 118) | 97(71, 129) | 85(68, 116) | 94.31(69, 115) | 0.430 |
| Triglycerides,mg/dL,median(Q1,Q3) | 119(87, 176) | 73.5(56.5, 91) | 110(92, 127) | 144(114, 180) | 223(171, 303) | <0.001 |
| Cholesterol,mg/dL,median(Q1,Q3) | 164(132, 194) | 155(122, 178) | 164.04(135, 192) | 157(131, 190) | 174(136, 205) | 0.011 |
| BUN,mg/dL,median(Q1,Q3) | 18(14, 24) | 17(13.5, 22) | 18(14, 26) | 17(13, 25) | 19(15, 26) | 0.225 |
| Glucose,mg/dL, mean±sd | 155.81±75.03 | 114.26±24.83 | 133.43±36.66 | 157.17±60.6 | 218±104.22 | <0.001 |
| max_bun,mg/dL,median(Q1,Q3) | 22(17, 35) | 20(16, 28.5) | 21(17, 41) | 22(17, 32) | 27(18, 55) | 0.004 |
| max_creatinine,mg/Dl,median(Q1,Q3) | 1.1(0.9, 1.6) | 1(0.8, 1.4) | 1.1(0.9, 1.5) | 1.1(0.9, 1.4) | 1.3(0.9, 2.5) | 0.002 |
| Statin | 424(97.47) | 106(98.15) | 105(96.33) | 108(99.08) | 105(96.33) | 0.475 |
| Antiplatelet | 434(99.77) | 108(100) | 109(100) | 108(99.08) | 109(100) | 0.392 |
| AKI score after PCI |  |  |  |  |  | . |
| 0 | 310(71.26) | 85(78.7) | 77(70.64) | 87(79.82) | 61(55.96) | 0.005 |
| 1 | 74(17.01) | 18(16.67) | 18(16.51) | 11(10.09) | 27(24.77) | . |
| 2 | 21(4.83) | 2(1.85) | 7(6.42) | 4(3.67) | 8(7.34) | . |
| 3 | 30(6.9) | 3(2.78) | 7(6.42) | 7(6.42) | 13(11.93) | . |
