# Supplementary material for: Association between the triglyceride–glucose index and acute kidney injury in patients undergoing percutaneous coronary: a retrospective analysis of the MIMIC-IV database
Source: Diabetol Metab Syndr. 2025 Mar 3;17:74. doi: 10.1186/s13098-025-01647-2 (PMC11874802; doi:10.1186/s13098-025-01647-2)
Supplement: Supplementary file 2 — Supplementary Material 2. [file 13098_2025_1647_MOESM2_ESM.docx]

# STable 2. Baseline characteristics by DM history

| **Characteristics** | **Overall**  **N=435** | **DM**  **N=138** | **Non DM**  **N=297** | **P value** |
| --- | --- | --- | --- | --- |
| TyG, mean±sd | 9.11±0.71 | 9.42±0.76 | 8.96±0.64 | <0.001 |
| Q1 | 108(24.83) | 12(8.7) | 96(32.32) | <0.001 |
| Q2 | 109(25.06) | 32(23.19) | 77(25.93) | . |
| Q3 | 109(25.06) | 39(28.26) | 70(23.57) | . |
| Q4 | 109(25.06) | 55(39.86) | 54(18.18) | . |
| Age, mean±sd | 67.14±13.72 | 70.64±12.5 | 65.52±13.97 | <0.001 |
| Sex |  |  |  |  |
| Male | 291(66.9) | 83(60.14) | 208(70.03) | 0.041 |
| Female | 144(33.1) | 55(39.86) | 89(29.97) | . |
| BMI,kg/m^2^, mean±sd | 28.76±5.76 | 29.52±6.2 | 28.4±5.53 | 0.059 |
| Sbp,mmHg, mean±sd | 125.73±21.69 | 128.71±23.69 | 124.35±20.59 | 0.051 |
| Dbp,mmHg, mean±sd | 73.42±16.15 | 69.48±16.03 | 75.25±15.9 | <0.001 |
| Hypertension | 296(68.05) | 112(81.16) | 184(61.95) | <0.001 |
| Diabetes | 138(31.72) | 138(100) | 0(0) | <0.001 |
| COPD | 34(7.82) | 16(11.59) | 18(6.06) | 0.045 |
| Peripheral vascular disease | 9(2.07) | 7(5.07) | 2(0.67) | 0.003 |
| Stroke | 18(4.14) | 12(8.7) | 6(2.02) | 0.001 |
| Cerebral hemorrhage | 3(0.69) | 3(2.17) | 0(0) | 0.011 |
| Sodium,mEq/L, mean±sd | 137.93±3.47 | 137.22±3.97 | 138.26±3.17 | 0.004 |
| Potassium,mEq/L,median(Q1,Q3) | 4.1(3.8, 4.4) | 4.2(3.9, 4.5) | 4.1(3.8, 4.4) | 0.088 |
| Calcium,mg/dL, mean±sd | 8.65±0.72 | 8.75±0.73 | 8.6±0.72 | 0.044 |
| Phosphate,mg/dL, mean±sd | 3.54±1.03 | 3.84±1.29 | 3.4±0.86 | <0.001 |
| Ast,IU/L,median(Q1,Q3) | 92.33(52, 160) | 68.42(41, 135.97) | 104.36(64, 171) | <0.001 |
| Alt,IU/L,median(Q1,Q3) | 42.86(28.46, 59) | 36.56(23, 56.12) | 44.75(31, 59.64) | 0.002 |
| Hemoglobin,g/dL, mean±sd | 12.64±2.07 | 12±2.11 | 12.93±1.99 | <0.001 |
| Platelet,K/uL, mean±sd | 233.01±81.66 | 234.28±97.17 | 232.42±73.53 | 0.826 |
| Wbc,K/uL,median(Q1,Q3) | 10.8(8.4, 13.9) | 10.7(8, 13.3) | 10.9(8.4, 14) | 0.374 |
| Creatinine,mg/dL,median(Q1,Q3) | 1(0.8, 1.2) | 1.1(0.8, 1.6) | 0.9(0.8, 1.1) | <0.001 |
| HDL,mg/dL,median(Q1,Q3) | 41.72(34, 50) | 40(28, 46) | 42(36, 51) | <0.001 |
| LDL,mg/dL,median(Q1,Q3) | 92(66, 118) | 78.82(53, 105) | 96(74, 124) | <0.001 |
| Triglycerides,mg/dL,median(Q1,Q3) | 119(87, 176) | 129.5(92, 195) | 115(86, 167) | 0.026 |
| Cholesterol,mg/dL,median(Q1,Q3) | 164(132, 194) | 144(116, 187) | 169(140, 198) | <0.001 |
| BUN,mg/dL,median(Q1,Q3) | 18(14, 24) | 22(16, 34) | 17(14, 21) | <0.001 |
| Glucose,mg/dL, mean±sd | 155.81±75.03 | 197.02±93.27 | 136.66±55.4 | <0.001 |
| max_bun,mg/dL,median(Q1,Q3) | 22(17, 35) | 33.5(21, 57) | 20(16, 27) | <0.001 |
| max_creatinine,mg/Dl,median(Q1,Q3) | 1.1(0.9, 1.6) | 1.4(1, 2.3) | 1(0.9, 1.3) | <0.001 |
| Statin | 424(97.47) | 135(97.83) | 289(97.31) | 0.748 |
| Antiplatelet | 434(99.77) | 138(100) | 296(99.66) | 0.495 |
| AKI score after PCI |  |  |  |  |
| 0 | 310(71.26) | 73(52.9) | 237(79.8) | <0.001 |
| 1 | 74(17.01) | 38(27.54) | 36(12.12) | . |
| 2 | 21(4.83) | 11(7.97) | 10(3.37) | . |
| 3 | 30(6.9) | 16(11.59) | 14(4.71) | . |
